# Fistulizing, Lower Esophageal Squamous Cell Carcinoma After Heller Myotomy With Fundoplication in a Patient With Achalasia

**DOI:** 10.14309/crj.0000000000001923

**Published:** 2025-12-05

**Authors:** Danzhu Zhao, Karmen Brar, Akaash Mittal, Haleh Vaziri

**Affiliations:** 1 Department of Medicine, University of Connecticut, Farmington, CT; 2 Division of Gastroenterology and Hepatology, Department of Medicine, University of Connecticut, Farmington, CT

**Keywords:** achalasia, esophagus, squamous cell carcinoma, heller myotomy, fundoplication

## Abstract

Achalasia is a premalignant condition with higher risk of esophageal squamous cell carcinoma (ESCC), but there is a lack of consensus on surveillance protocol need because of inconsistent data regarding the increased risk of ESCC in these patients compared to the general population. ESCC is generally proximally located and less common than esophageal adenocarcinomas. Although most ESCC in patients with a history of achalasia are found in the middle third of the esophagus, patients who underwent surgical intervention resulting in a modified anatomy of the esophagus and the upper stomach may have a different presentation and endoscopic findings. Unfortunately, the diagnosis of ESCC may be delayed as symptoms of achalasia and esophageal carcinomas may overlap. To our knowledge, this case represents the first reported instance of a patient with distal ESCC with fistulization to the upper stomach because of a prior Heller myotomy and fundoplication for achalasia.

## INTRODUCTION

Achalasia is a known risk factor for esophageal carcinomas, including both esophageal squamous cell carcinoma (ESCC) and esophageal adenocarcinoma.^1^ ESCC is more prevalent in Asian countries and commonly associated with alcohol consumption, tobacco use, as well as caustic exposure such as hot beverages.^1^ ESCC is most commonly located in the middle third of the esophagus, whereas esophageal adenocarcinoma more commonly presents in the lower third of the esophagus and gastroesophageal (GE) junction.^2^ Studies have demonstrated that patients with achalasia have over 10 times increased risk of ESCC than the general population.^3^ Patients with a history of achalasia and development of ESCC may have overlapping symptoms that may delay the diagnosis of malignancy. We present a case of a patient who was found to have lower ESCC with fistulization to the upper stomach but sparing the GE junction, because of prior Heller myotomy (HM) and fundoplication for treatment of achalasia. Our case highlights the potential long-term complications of achalasia even after treatment, and the potential utility of cancer surveillance in these high-risk individuals who may have overlapping symptoms of achalasia and ESCC.

## CASE REPORT

**Figure 1.**
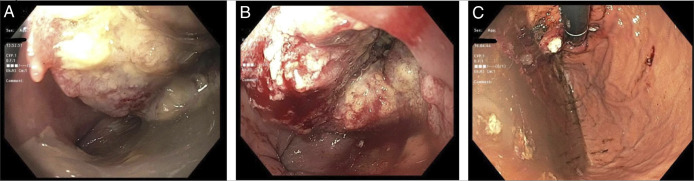
(A) A large, fungating mass about 4 cm above the Z line. The mass was nonobstructing and partially circumferential (involving one-third of the lumen circumference). (B) There was fistula-like tract with the opening in the middle of the mass. (C) A fungating mass was found in the gastric fundus mostly covered by food.

A 55-year-old White woman with a history of achalasia and HM with fundoplication (unknown type) 7 years before presentation who was brought to the emergency department with subacute headache and right arm weakness. She had dysphagia for the past few months with worsening regurgitation more recently despite proton pump inhibitor use. The patient did not follow up with her gastroenterologist as she thought her symptoms were related to her achalasia. She consumed minimal alcohol and denied tobacco use. Her vital signs, physical examination, and labs were unremarkable. Brain imaging revealed a 3.3 cm by 3.2 cm left parietal lobe mass. Computed tomography (CT) chest, abdomen, and pelvis showed an inferior mediastinal mass originating from either the herniated portion of gastric fundus or distal esophagus, although the former was favored. Invasion to liver and metastatic lesions to bone and adrenal glands were also noted. Upper endoscopy (EGD) revealed a large, fungating mass 4 cm above the Z line with a fistula-like tract emanating from the middle of the mass (Figure 1). Retroflexion showed a fungating mass partially covered by food (Figure 1). Mass biopsies revealed invasive ESCC. Biopsies stained positive for programmed death ligand-1 (PDL-1), and she was ultimately initiated on anti-programmed cell death protein-1 (anti–PD-1) along with folinic acid, fluorouracil, and oxaliplatin. She was discharged with gastroenterology (GI), oncology, and neurosurgery follow-up.

**Figure 2.**
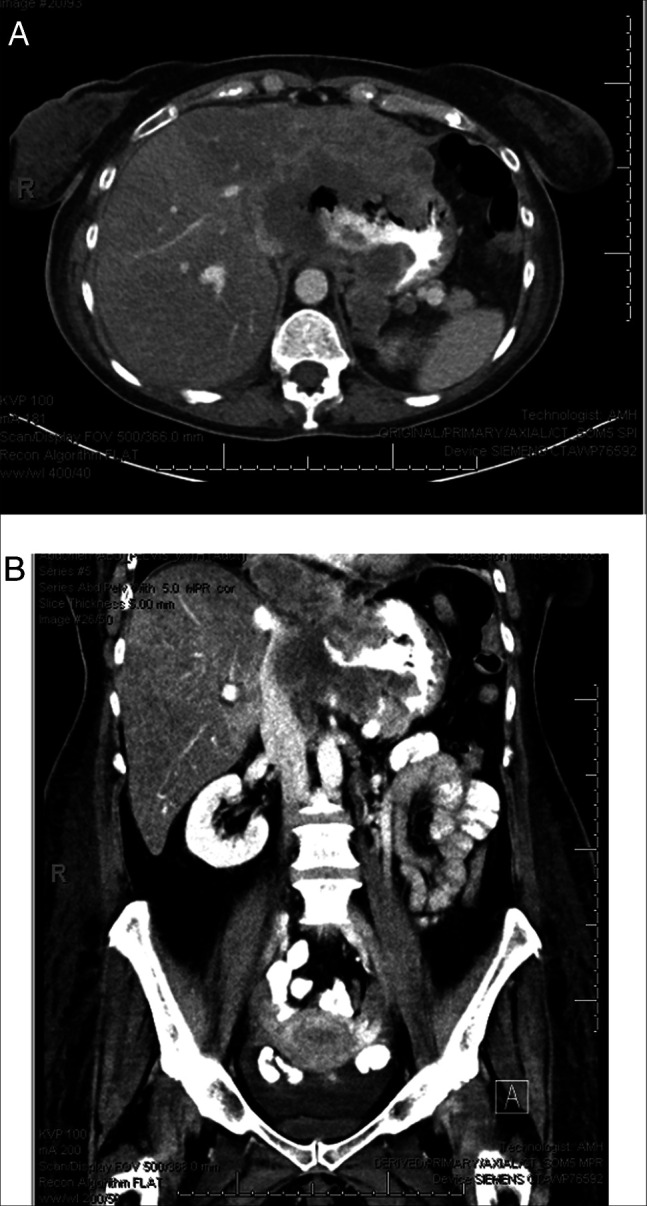
(A) CT image axial view of the enlargement of the GE junction/gastric fundus malignancy with central necrosis in addition to oral contrast filled channels extending rightward from the distal esophagus to the gastric fundus. (B) CT image coronal view of the enlarged GE junction/gastric fundus malignancy with oral contrast extending from the distal esophagus to the gastric fundus. GE, gastroesophageal.

She unfortunately required a few rehospitalizations after her initial discharge. She developed a left parietal lobe abscess and *Micrococcus luteus* bacteremia, necessitating a left parietal craniotomy and an extended course of treatment with intravenous antibiotics. Concurrently, she developed progressive dysphagia and abdominal pain, prompting a repeat abdominal CT scan 2 months after her initial diagnosis of ESCC to reassess the disease burden. Imaging demonstrated marked enlargement of the GE junction/gastric fundus malignancy with central necrosis in addition to oral contrast filled channels extending rightward from the distal esophagus to the gastric fundus (Figure 2). In addition, new left hepatic lobe metastases, progressive upper abdominal and retroperitoneal adenopathy, tumor encasement with vascular compression of multiple upper abdominal vessels, enlargement of adrenal mass, and ascites were noted. Given her nutritional needs and extent of disease progression, the patient initially underwent percutaneous endoscopic gastrostomy tube placement for enteral nutrition but ultimately elected to forgo further oncologic therapy, transitioning to hospice care.

## DISCUSSION

Achalasia is a rare esophageal motility disorder that develops because of degeneration of the ganglion cells in the myenteric plexus of the esophagus, leading to reduced lower esophageal sphincter relaxation and loss of peristalsis.^2,4^ It is a potential risk factor for esophageal carcinoma, particularly ESCC. Long-standing or suboptimal treatment of achalasia may cause stasis and slow elimination of foods, bacterial overgrowth, and prolonged acid exposure to the esophageal mucosa with reflux symptoms.^2^ This creates an environment prone to chronic inflammation and damage to the esophageal mucosa, ultimately increasing the risk for hyperplastic changes and malignant transformation with time.^5–7^ Studies have also suggested increased frequency of p53 overexpression in patients with achalasia, potentially increasing the risk of ESCC.^8,9^

Although ESCC predominantly arises in the mid-esophagus, the tumor in our case was in the distal third of the esophagus, a region more commonly associated with Barrett's esophagus and adenocarcinoma.^10^ Interestingly, the esophageal mass invaded the upper stomach without involving the GE junction, likely because of surgically altered anatomy, making this a distinct presentation. This likely resulted from the patient's previous HM and fundoplication that raised her upper stomach adjacent to the involved esophageal area years earlier, altering the anatomical alignment of the distal esophagus and upper stomach. The patient did not have any other classically identifiable risk factors for developing ESCC, thus highlighting the importance of monitoring patients with history of achalasia regardless of the type of intervention they have received. Patients with achalasia may alternatively undergo per oral esophageal myotomy (POEM) because of its less invasive approach and similar long-term success as HM.^11^ The higher risk of ESCC in achalasia has also been demonstrated in patients who had undergone POEM.^12^ Shiwaku et al identified 3 subtypes of achalasia including straight, sigmoid and advanced sigmoid achalasia with no statistically significance difference in the incidence of esophageal cancer between straight and sigmoid types after undergoing POEM (*P* = 0.36).^12^ Persistent or recurrent symptoms despite previous interventions to treat achalasia warrant reevaluation as these interventions do not completely preclude the risk for malignant transformation. Our patient presented with dysphagia, but prior fundoplication may have affected the spread of tumor cells given the altered esophageal and upper stomach anatomy. We believe that patients who have undergone myotomy and fundoplication should be considered for further testing not only when they have recurrence of dysphagia but also with abdominal pain or fullness as these symptoms may result from gastric involvement by esophageal cancer. The upper stomach should be evaluated carefully in these patients during retroflexed view.

Diagnosing ESCC in the context of achalasia poses a significant challenge because of overlapping symptoms, such as dysphagia, regurgitation, and weight loss. These similarities often delay recognition of malignancies. Delay in diagnosis may also be attributed to challenges with endoscopic surveillance of achalasia because of poor visualization from retained food.

Moreover, the absence of standardized guidelines for cancer screening further complicates early detection. The 2020 American College of Gastroenterology guidelines recommend against routine endoscopic cancer screening in patients with achalasia because of limited evidence, although expert opinions suggest surveillance every 3 years for individuals with more than a decade of disease.^13^ This case underscores the need for heightened index of suspicion and monitoring in patients with achalasia, regardless of a history of surgical interventions and the importance of individualized surveillance strategies, especially in patients with persistent or worsening symptoms despite prior interventions. Given the conflicting data on ESCC in achalasia resulting in the lack of consensus on screening protocol, further research is needed to evaluate the utility of long-term cancer surveillance and refine risk stratification for patients with achalasia. Although current evidence remains limited, a tailored approach to surveillance, based on presence of symptoms, symptom progression, disease duration, and treatment history, may improve early detection and outcomes in this high-risk population.^7,13^ Persistent or recurrent dysphagia despite prior achalasia interventions warrants timely endoscopic reevaluation. Altered anatomy because of prior achalasia interventions may influence both tumor presentation and surveillance approach.

## DISCLOSURES

Author contributions: D. Zhao: drafted the manuscript, edited, approved of publication, and accountable for all aspects of the manuscript. K. Brar: assisted in drafting the manuscript, edited, and approved of publication. A. Mittal: assisted in drafting the manuscript, edited, and approved of publication. H. Vaziri: substantially contributed to the conception of the manuscript, assisted in drafting the manuscript, edited, approved of publication, and is the article guarantor.

Financial disclosure: None to report.

Previous presentation: ACG 2024 Annual Scientific Meeting and Postgraduate Course; October 25–30, 2024; Philadelphia, Pennsylvania. Abstract P2241.

Informed consent could not be obtained for this case report. All identifying information has been removed.

## ABBREVIATIONS:

anti-PD-1: anti-programmed cell death protein -1
CT: computed tomography
EGD: endoscopy
ESCC: esophageal squamous cell carcinoma
GE: gastroesophageal
GI: gastroenterology
HM: Heller myotomy
PDL-1: programmed death ligand -1
POEM: per oral esophageal myotomy
